# The Effects of Canopy Height and Bud Light Exposure on the Early Stages of Flower Development in *Prunus persica* (L.) Batsch

**DOI:** 10.3390/plants9091073

**Published:** 2020-08-20

**Authors:** Madeleine Peavey, Ian Goodwin, Lexie McClymont

**Affiliations:** Agriculture Victoria, Tatura, VIC 3616, Australia; ian.goodwin@agriculture.vic.gov.au (I.G.); lexie.mcclymont@agriculture.vic.gov.au (L.M.)

**Keywords:** bud shading, floral bud, fruit set, leaf pluck, nectarine, photosynthetically active radiation

## Abstract

The aims of this study were to investigate the sunlight requirements during floral initiation and differentiation for the development of flower buds in ‘Autumn Bright’ nectarine and to explore its source–sink relationship. In early January 2019 (111 days after full bloom), prior to floral initiation and differentiation, 12 new shoots were tagged on 14 trees, with four shoots in each of the low (0–1.2 m), middle (1.2–2.0 m), and high (>2.0 m) canopy heights. Three treatments (bud shading; leaf pluck; bud shading and leaf pluck) were applied to three shoots in each canopy height on the fourth and eighth bud, in addition to a fourth control shoot. Light penetration was measured at the different canopy heights. Buds were assessed in Spring for floral transition, number of floral buds per node, and fruit set. The treatments at the node level had no effect on floral initiation, indicating that sink strength was not promoted by additional light. Light penetration decreased with decreasing canopy height and corresponded with lower floral buds in the low zone. Fruit set was uninfluenced by all treatments. The results of this study emphasised the importance of the availability of photosynthetic assimilates for floral initiation in peach and nectarine trees. Balanced crop load management and summer pruning to enhance canopy sunlight distribution would increase the availability of nutrients for improved floral transition in this cultivar.

## 1. Introduction

Peach and nectarine (*Prunus persica* L. Batsch) are globally important crops, with a combined international production of 22.3 million tonnes in 2019 [1]. The main producers are China, the European Union, Turkey, and the United States. Australian production of peach and nectarine has been increasing in recent years to 96,000 tonnes due to orchard replanting with higher-yielding cultivars [2]. The vast majority (77%) of peach/nectarine crops are grown in the state of Victoria, in the nation’s southeast. The popularity of peach and nectarine amongst Australian consumers and Australian export markets calls for consistency and/or improvements in yield. A primary indicator of the yield potential of a temperate tree crop is the quantity of flowers formed from overwintering floral buds. Maintaining high production is important for commercial growers, and therefore, gaining knowledge in methods to manipulate and improve flower morphogenesis is relevant. This is important to improve the distribution of flowers throughout the tree canopy and avoid the development of barren wood. Potentially, flower location could be better placed for more convenient and economic management (i.e., flower and fruit thinning, harvesting, and spraying).

Of central importance to this study are the early stages of flower development, namely floral initiation and differentiation, which are respectively defined as all the necessary developments required for commitment by the meristem to create an inflorescence [3] and the morphological changes to the meristem to irreversibly form a flower [4]. These initial steps are triggered differently between species, with some flowers developing entirely autonomously and others relying on environmental stimuli to begin the transition to creating a flower [5]. Often, the control of floral initiation results from an interaction between endogenous factors, namely genetic and hormonal signalling systems (i.e., gibberellin), and environmental factors [5]. The study of floral development in temperate tree crops has been varied across different species, with long reproductive cycles and incomplete genome sequencing presenting barriers to in-depth research. The temperature and photoperiod (day length) during the floral initiation and differentiation stages are important environmental factors influencing floral development, but solar irradiance has also been shown to play a positive part [4,5,6,7,8]. Solar irradiance can affect floral morphogenesis by influencing the photosynthetic capacity of the source leaves or at the sink (the undifferentiated bud) by promoting its strength [8,9]; however, these effects have not been directly associated with the stages of floral initiation and differentiation. From this statement stems the question as to whether direct light exposure—and concomitantly higher bud temperature—can influence the chemical pathways involved in floral initiation and differentiation. It has been suggested that for citrus, cold temperatures may be perceived by the stem or buds, stimulating the induction of flowering [10]. It is not known if the undifferentiated meristem of peach and nectarine trees responds directly to light and/or temperature.

The period of floral initiation in peach and nectarine trees has been determined as beginning in late January in the southern hemisphere in a temperate climate [11,12]. Peach floral and vegetative buds form laterally in the axils of leaves on the current season’s growth [6]. Flower bud distribution varies between peach and nectarine cultivars, but a vegetative bud is most often flanked by two floral buds [4]. In some cultivars, there can be up to three floral buds per node, and each floral bud can set up to four fruits. This is known as “fruit doubling”, and the cause is thought to be heat and water stress during floral initiation and differentiation, leading to the development of multiple pistils in *Prunus* species [13,14].

The aim of this study was to investigate the importance of sunlight during floral initiation and differentiation for the development of flower buds in nectarine. The effects of canopy height and direct bud shading and leaf pluck during floral initiation and differentiation were examined, and the source–sink relationship of these stages of flower development in peach and nectarine is discussed.

## 2. Results

### 2.1. Node Type and Floral Bud Counts

The overall proportions of floral and vegetative nodes within the control treatment (including all canopy height zones) were 49% floral and 51% vegetative (Table 1). Similarly, the percentage of vegetative nodes remained higher than that of floral nodes amongst all node treatments and canopy heights.

Pearson’s chi-square tests were performed to examine the relationships between node type and canopy height, and between node type and node treatment. The relationship between node type and canopy height was significant (*Χ*^2^, *p* = 0.001) with the proportion of floral nodes increasing with increasing height above the ground (Table 1). The percentage of floral nodes situated in the low canopy zone was particularly affected, with reductions of 42% and 50% from the middle and high zones, respectively. The relationship between node type and node treatment showed no association (*Χ*^2^, *p* = 0.143; Table 1), despite there being a tendency for a reduction in floral node percentage from the control.

Pearson’s chi-square tests between *fbud_node_* and canopy height, and between *fbud_node_* and node treatment likewise found no significant associations for either factor (*Χ*^2^, *p* = 0.114 and *p* = 0.564, respectively; Table 2). Of the total floral nodes, 61% contained one floral bud, 38% contained two, and 1% contained three (Table 2). There was a trend for the number of single budded nodes to decrease with increasing height, while the number of double budded nodes increased.

### 2.2. Fruit Set

Most sample floral nodes set either one or two fruits (43% and 28%, respectively; Table 3). Pearson’s chi-square tests were performed to examine the relationships between *fs_node_* and canopy height, and between *fs_node_* and node treatment. Neither factor had significant associations (Χ^2^, *p* = 0.141 and *p* = 0.219, respectively; Table 3). The middle canopy height represented the highest *fs_fbud_* value (1.34), and the lowest value was found in the low canopy height (1.06), although there was no significant correlation (*p* = 0.213; Table 4). There was also no association between node treatment and *fs_fbud_* (*p* = 0.053; Table 4).

### 2.3. Light Penetration

**Figure 1:**
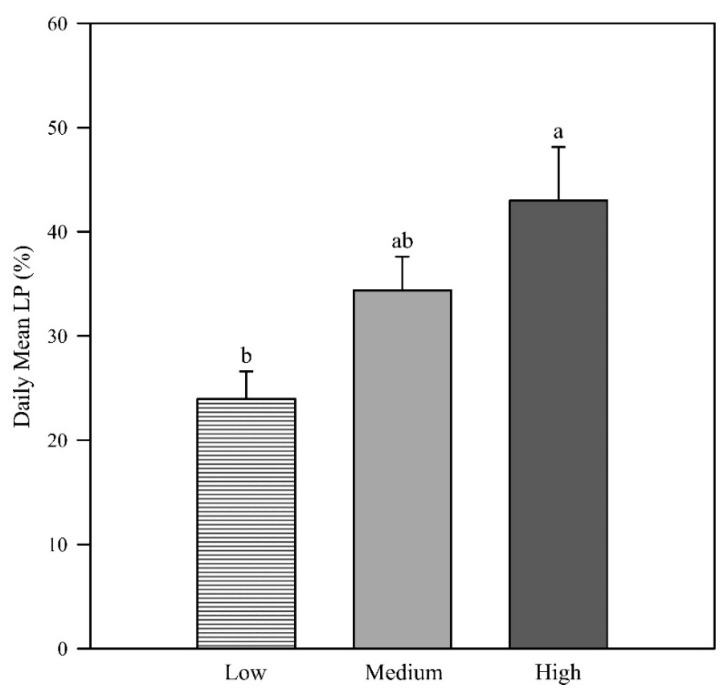
The daily mean light penetration at low, middle, and high canopy zones (*p* = 0.005). Error bars represent the standard errors of the means. Letters represent the mean separation of data by Fisher’s unprotected LSD (α = 0.05).

**Figure 2:**
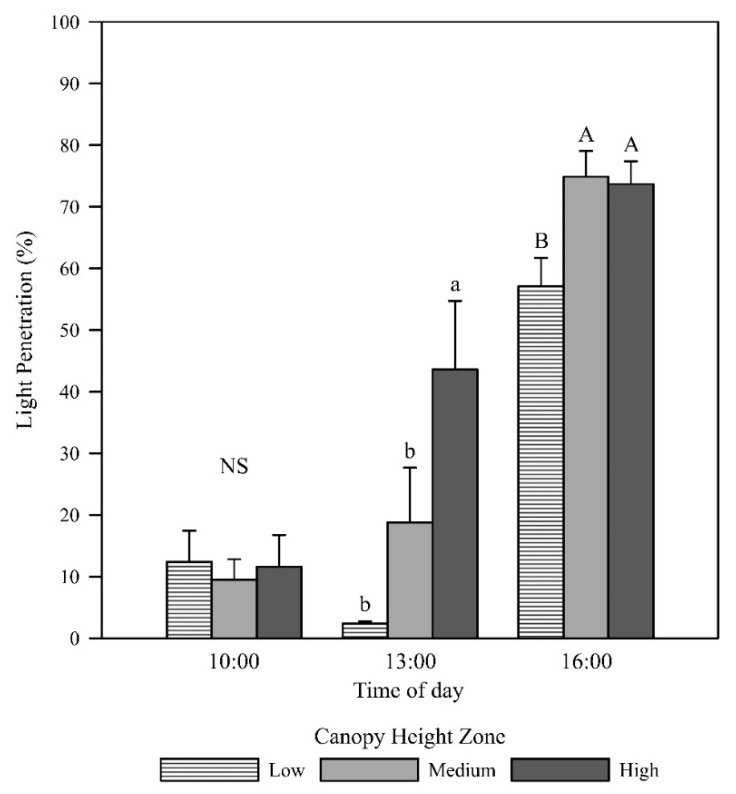
Mean light penetration at low, middle, and high canopy zones taken in the morning (10:00 h AEST; *p* = 0.899), at solar noon (13:00 AEST; *p* = 0.001), and in the afternoon (16:00 AEST; *p* = 0.014). Error bars represent the standard errors of the means. Letters represent mean separation of data at each time point by Fisher’s unprotected LSD (α = 0.05).

Daily mean light penetration increased with increasing canopy height above the ground, with the high zone displaying significantly higher light penetration than the low zone (*p* = 0.005; Figure 1). In the morning, light penetration was low at all canopy heights (low zone = 12.4%, medium zone = 9.5%, high zone = 11.6%) with no difference in the group means (*p* = 0.899; Figure 2). This result is likely due to the measurements having been taken in the western canopy, resulting in more depth of canopy for the sunlight to penetrate. The photosynthetic photon flux density (PPFD) readings at solar noon and in the afternoon revealed that the low canopy zone had the least light penetration (2.4% and 57.1%, respectively). At solar noon, the high zone had significantly higher light penetration (43.6%) than the low and middle zones (*p* = 0.001), and in the afternoon, the light penetration of the middle and high zones (74.9% and 73.7%) was significantly higher than in the low zone (*p* = 0.014).

## 3. Discussion

The results of better daily light penetration at higher canopy heights and the enhanced transition to floral nodes within these zones indicate a positive relationship between the quantity of sunlight and floral initiation and differentiation. The minimum percentage of full sun required for the transition to a floral bud has previously been quantified for apple using hemispherical photography [15]; however, no clear distinction was made between the light exposure of the leaves as opposed to the buds. It would be possible to quantify an irradiance or daily light integral threshold for floral transition in nectarine (or other species) with in situ radiation sensors, providing a more intensive and precise assessment of light penetration at the nodal level. The lack of effect from the node shading treatments indicates that more irradiance exposure does not strengthen the undifferentiated meristem as a sink for photosynthates and hormones responsible for floral induction. This is contrary to what has been demonstrated by May [16] for grapevines, where the shading of individual buds, in a similar manner to our study, reduced the transition of undifferentiated buds to become floral by 30%. The same effect has not been apparent in perennial horticultural trees. In most cases with fruit trees, shading of the whole tree, or part of the tree, has resulted in a decrease in flower bud formation [6,17,18,19,20,21]. This is indicative of the significance of photosynthetic assimilates in the floral transition in these species, and the evidence in this study is in agreement. Although there was no effect on floral development from removing the node leaves adjacent to the undifferentiated sample buds, the proximity of source leaves to the undifferentiated bud may still be implicated judging by the apparent effect of the canopy height zones. Future studies into peach floral relationships with sunlight need more focus on the individual stages of initiation and differentiation, canopy shading, and light penetration.

Studies in fruit trees, including peach, have documented the negative effect of a high current season’s crop load on floral initiation due to the reduced availability of photosynthates for floral transition [22,23,24]. There was no available crop load data for the sample trees in this study, although it is unlikely that a high fruit load in the low zone compared to the middle and high zones can be the cause of the reduced floral bud count.

Therefore, it is proposed that the modern cultural management of peach and nectarine trees to improve floral initiation and differentiation should concentrate on refining crop load limits and canopy sunlight distribution in order to optimise photosynthate availability in all areas of the tree. This is usually undertaken through various methods of chemical or manual fruit thinning and winter and summer pruning techniques. According to Jackson [25], optimising light distribution throughout the tree canopy maximises the efficiency of light utilisation in flower bud formation. Therefore, it is worth considering the timing of summer pruning with respect to the periods of floral initiation and differentiation, and the severity of pruning, which would alter canopy light penetration to varying degrees and the total area of photosynthesising leaves. Notably, net photosynthesis and transpiration in the basal parts of peach and apple trees improved with severe summer pruning [26]. In sweet cherry, summer pruning one-year-old shoots 15 to 30 cm from the shoot base improved flower bud differentiation, as it removed the influence of the apical meristem [27]. Summer pruning of the trees in this study may have improved floral initiation and differentiation in the lower zones, and the results demonstrate what can occur without it.

While temperature was not considered a factor in this experiment, it could be important in peach floral initiation and differentiation and it would be interesting to assess for any bud or canopy temperature differences at the various canopy heights. The temperature of a bud or leaf could be altered by the ambient air temperature or through radiant heating from direct light exposure. Consequently, the higher solar irradiance present in the upper zones of the tree could lead to higher temperatures, which may be implicated in enhancing flower bud development through the stimulation of hormonal pathways. Since floral initiation in peach and nectarine occurs in the middle of summer, proximity to the ground could provide a warming effect to the lower zones of the canopy. A study into the canopy temperature of peach trees grown in a spindle bush system by Septar et al. [28] did not find significant differences in the temperature of a canopy divided into five zones, although there was a tendency for a slight increase at the base of the canopy (0.1–0.3 °C). A similar experiment performed on a vertical, two-dimensional canopy, such as our trees, may yield different results.

There is a gap in the literature on the relationship between light exposure (whether that be source leaves or the sink bud) during the floral initiation and differentiation periods and flower bud strength and fertility in peach. Nuzzo et al. [8] discovered that seasonal light availability in the upper versus the lower part of the canopy of *Prunus armeniaca* L. had a positive relationship with flower bud nutrition and pistil development. However, the effects in that study were not differentiated by light exposure at various stages of flower development. While there were no effects from the treatments in this study on fruit set and fruit doubling, there is space for further research into peach flower bud quality as affected by light availability during floral initiation and differentiation.

## 4. Materials and Methods

### 4.1. Study Site and Plant Material

The experiment was conducted in the Stone Fruit Experimental Orchard located at Agriculture Victoria, Tatura (Victoria, Australia; 36.435° S 145.270° E) over two consecutive seasons from 2018 to 2020 (2018–2019 and 2019–2020 in the southern hemisphere). The trees were six-year-old ‘Autumn Bright’ nectarine (*Prunus persica* (L.) Batsch var. *nucipersica*), grafted onto Nemaguard rootstock, and trained in a two-leader bi-axis system on a vertical trellis oriented north–south. This cultivar blooms in the Goulburn Valley in early to mid-September, sets fruit by mid-October and is harvested in late January to early February. Chilling requirement is approximately 650 h (0–7.2 °C Model) [29,30]. The top wire of the trellis was situated at 2.4 m above ground level, and the tops of the trees extended approximately 40 cm above the top wire. Row and tree spacings were 4.5 and 1 m, respectively. Measurement trees were not summer pruned; however, adjacent non-sample trees were summer pruned. Measurement trees were drip irrigated.

### 4.2. Treatments and Experimental Design

Two independent factors were investigated in this experiment, and for the purposes of this study, they are named ‘Canopy Height’ and ‘Node Treatment’. Fourteen measurement trees were chosen that were distributed throughout a single row in the orchard.

#### 4.2.1. Canopy Height

The canopy of each tree was divided into three heights: a low zone from ground level to 1.2 m, a middle zone from 1.2 to 2.0 m, and a high zone from 2.0 m and above. Within each height zone, four new shoots of approximately 30 cm length and on the west side of the canopy were chosen and tagged. The buds of the fourth and eighth nodes starting from the base of each shoot were marked nearby with a permanent marker. The fourth node was located on the basal-median segment of the shoot and the eighth node was located on the apical-median segment.

#### 4.2.2. Node Treatments

At 111 days after full bloom, treatments were applied to both the sample nodes (fourth and eighth) on the tagged shoots in each height zone. The treatments were bud shading (BS), leaf pluck (LP), bud shading in combination with leaf pluck (BS/LP), and control (C). Aluminium hoods were secured around BS and BS/LP sample nodes to achieve shading from direct sunlight, with an opening underneath to allow, to prevent temperature increases above that of the external environment and to allow the entrance of diffuse light. The buds were therefore not exposed to direct sunlight at any time of the day. Shaded buds of the BS treatment maintained their leaves, which were left protruding from the opening of the aluminium shade. Buds of the LP and BS/LP treatments had their foliage removed. Control buds were left unshaded and with foliage. Aluminium shades were removed from all BS and BS/LP buds after leaf fall to mimic heavy canopy shading throughout the stages of floral initiation and differentiation. The buds of 26 nodes were mechanically damaged and were removed from the overall sample (*n* = 310).

### 4.3. Measurements

At BBCH stage 51 (bud swell) [31], a count was done on the sample nodes firstly to categorise them as floral or vegetative, and secondly, to count the number of floral buds per sample floral node (*fbud_node_*). A floral node was determined as having at least one floral bud. Floral buds were distinguished from vegetative buds based on several visual features. Vegetative buds are conically shaped, smaller, and covered with a fine layer of trichomes, whereas floral buds are globe shaped, larger, and covered with a more intense trichome layer [4].

A count for the number of fruit set for each sample floral node (*fs_node_*) was performed at BBCH stage 73 (31 days after full bloom [DAFB]). The number of fruit set per floral bud (*fs_fbud_*) was calculated with the formula:

$$fs_{fbud}=\frac{fs_{node}}{fbud_{node}}.$$

An *fs_fbud_* value of greater than or equal to 2 indicates fruit doubling.

Photosynthetically active radiation (PAR) was quantified as photosynthetic photon flux density (PPFD) using a Sunfleck PAR ceptometer (Decagon Devices Inc., Pullman, WA, USA) in the morning (10:00 h AEST), at solar noon (13:00 h AEST), and in the afternoon (16:00 h AEST) during January. The waveband of light utilised by green plants for photosynthesis lies from 400 to 700 nm and is aptly named photosynthetically active radiation (PAR) [32]. The ceptometer was positioned horizontally spanning across the west side of sample trees and offset to be situated within the canopy approximately 20 cm away from the trunk/leaders. Four readings were taken at each sample tree: one reference reading in full sun (at 1.6 m above ground level) and one in the middle of each of the three canopy height zones (0.6 m; 1.6 m; 2.5 m). Light penetration (%) was calculated with the formula:

$$Light\;Penetration=\left(\frac{PPFD_{m}}{PPFD_{r}}\right)\times 100$$

where *PPFD_m_* was the within-canopy measurement and *PPFD_r_* was the reference measurement taken in full sun. The daily mean light penetration for each canopy height zone was calculated by taking the average from the morning, solar noon, and afternoon measurements.

### 4.4. Statistical Analyses

Nodal category (floral or vegetative), *fbud_node_*, and *fs_node_* were analysed by Pearson’s chi-square test using SYSTAT^®^ 13.1 (SYSTAT^®^ Software Inc., San Jose, CA, USA) statistical computing software, with factors of canopy height and node treatment assessed independently. Only floral nodes were utilised for the analysis of floral bud data. *fs_fbud_* and light penetration data were analysed using RCBD-based analysis of variance (ANOVA) using Genstat^®^ 19.1 (VSN International Ltd., England, UK) statistical computing software.

## 5. Conclusions

Higher quantities of sunlight present in the middle and upper zones of the canopy correlated with an increased transition to floral buds. However, direct sunlight exposure on undifferentiated buds compared to completely shaded buds did not influence floral initiation, indicating that sink strength was not promoted by light. A degree of proximity of undifferentiated buds to sunlight-exposed leaves likely plays a role in enhancing floral initiation through the provision of more readily available photoassimilates. This signifies that pruning or defoliation to increase bud light exposure would be redundant. Providing balanced crop load management and summer pruning to enhance canopy light penetration would be more useful in improving the availability of assimilates for floral transition in this cultivar and the subsequent season’s distribution of flowers and fruit in the tree canopy. More knowledge is required regarding the cultivar-specific timing and severity of summer pruning for the enhancing floral initiation.

## Figures and Tables

**Table 1:** The percentage of floral and vegetative nodes for each canopy height zone (low, middle, high) and for each node treatment (BS = bud shading; LP = leaf pluck; BS/LP = bud shading plus leaf pluck; C = control). Pearson’s chi-square test with canopy height, *X*^2^ (2, *N* = 310) = 14.640, *p* = 0.001. Pearson’s chi-square test with node treatment, *X*^2^ (3, *N* = 310) = 5.425, *p* = 0.143.

Node Type	Canopy Height			Node Treatment			
	Low	Middle	High	BS	LP	BS/LP	C
Floral	25	43	50	41	34	32	49
Vegetative	75	57	50	59	66	68	51
*Total*	*100*	*100*	*100*	*100*	*100*	*100*	*100*

**Table 2:** The percentage of floral nodes with one, two, or three floral buds (*fbud_node_*) for each canopy height (low, middle, high) and for each node treatment (BS = bud shading; LP = leaf pluck; BS/LP = bud shading plus leaf pluck; C = control). Pearson’s chi-square test with canopy height, *X*^2^ (4, *N* = 122) = 7.443, *p* = 0.114. Pearson’s chi-square test with node treatment, *X*^2^ (6, *N* = 122) = 4.840, *p* = 0.564.

*fbud_node_*	Canopy Height			Node Treatment			
	Low	Middle	High	BS	LP	BS/LP	C
One	75	61	54	55	64	73	58
Two	21	39	46	42	36	27	42
Three	4	0	0	3	0	0	0
*Total*	*100*	*100*	*100*	*100*	*100*	*100*	*100*

**Table 3:** The percentage of floral nodes that set zero, one, two, three, four, or five fruit (*fs_node_*) for each canopy height (low, middle, high) and for each node treatment (BS = bud shading; LP = leaf pluck; BS/LP = bud shading plus leaf pluck; C = control). Pearson’s chi-square test with canopy height, *X*^2^ (10, *N* = 122) = 14.752, *p* = 0.141. Pearson’s chi-square test with node treatment, *X*^2^ (15, *N* = 122) = 18.885, *p* = 0.219.

*fs_node_*	Canopy Height			Node Treatment			
	Low	Middle	High	BS	LP	BS/LP	C
Zero	18	4	6	3	16	8	8
One	57	39	40	33	36	61	45
Two	11	30	35	27	28	27	29
Three	11	15	17	30	8	4	13
Four	3	7	2	3	8	0	5
Five	0	4	0	3	4	0	0
*Total*	*100*	*100*	*100*	*100*	*100*	*100*	*100*

**Table 4:** The mean number of fruit set per floral bud (*fs_fbud_*) for each canopy height (low, middle, high; *p* = 0.213) and for each node treatment (BS = bud shading; LP = leaf pluck; BS/LP = bud shading plus leaf pluck; C = control; *p* = 0.053). S.E. represents the standard errors of the means. *p* represents the probability value.

	Canopy Height			Node Treatment			
	Low	Middle	High	BS	LP	BS/LP	C
*fs_fbud_*	1.06	1.34	1.18	1.45	1.16	1.02	1.19
*S.E.*	*0.117*	*0.092*	*0.089*	*0.106*	*0.123*	*0.120*	*0.098*
*p*	*0.213*			*0.053*			
